# Exercise Training Improves the Altered Renin-Angiotensin System in the Rostral Ventrolateral Medulla of Hypertensive Rats

**DOI:** 10.1155/2016/7413963

**Published:** 2016-01-05

**Authors:** Chang-zhen Ren, Ya-Hong Yang, Jia-cen Sun, Zhao-Tang Wu, Ru-Wen Zhang, Du Shen, Yang-Kai Wang

**Affiliations:** Department of Physiology, Second Military Medical University, Shanghai 200433, China

## Abstract

The imbalance between angiotensin II (Ang II) and angiotensin 1–7 (Ang 1–7) in the brain has been reported to contribute to cardiovascular dysfunction in hypertension. Exercise training (ExT) is beneficial to hypertension and the mechanism is unclear. This study was aimed to determine if ExT improves hypertension via adjusting renin angiotensin system in cardiovascular centers including the rostral ventrolateral medulla (RVLM). Spontaneously hypertensive rats (SHR, 8 weeks old) were subjected to low-intensity ExT or kept sedentary (Sed) for 12 weeks. Blood pressure elevation coupled with increase in age was significantly decreased in SHR received ExT compared with Sed. The results in vivo showed that ExT significantly reduced or increased the cardiovascular responses to central application of sarthran (antagonist of Ang II) or A779 (antagonist of Ang 1–7), respectively. The protein expression of the Ang II acting receptor AT1R and the Ang 1–7 acting receptor Mas in the RVLM was significantly reduced and elevated in SHR following ExT, respectively. Moreover, production of reactive oxygen species in the RVLM was significantly decreased in SHR following ExT. The current data suggest that ExT improves hypertension via improving the balance of Ang II and Ang 1–7 and antioxidative stress at the level of RVLM.

## 1. Introduction

Hypertension is characterized by elevated levels of blood pressure (BP) and sympathetic tone, which are associated with the development and prognosis of this disease [1, 2]. It is well known that the rostral ventrolateral medulla (RVLM) is a key region for central control of sympathetic outflow and plays a vital role in maintaining resting BP and sympathetic tone [3]. It has been suggested that abnormalities in structure and function of the RVLM contribute to the pathogenesis of the neurogenic hypertension [4, 5].

It is also well known that the renin-angiotensin system (RAS) has widely cardiovascular regulatory effects and its abnormality participates the generation and development of hypertension [6, 7]. Besides the peripheral RAS as we know well, there is another independent RAS in the central nervous system, which also has been implicated in the pathogenesis of hypertension [8]. All of the components of the RAS (precursor, enzymes, peptides, and receptors) are presented in brain areas (e.g., RVLM, NTS, and PVN) involved in cardiovascular control [9]. In a classical RAS, angiotensin (Ang) II produced from Ang I by an angiotensin-converting enzyme (ACE) is a strong bioactive substance and its activation contributes to the development of the hypertension [10]. Besides the ACE-Ang II-AT_1_R axis, recent studies have established a new regulatory axis in the RAS [11]. In this axis, angiotensin (Ang) (1–7) is finally produced from Ang I or Ang II by the catalytic activity of angiotensin-converting enzyme 2 (ACE2). Ang (1–7) shows actions different from those of AT_1_R stimulation, such as vasodilatation, natriuresis, and antiproliferation [12]. It has been found that the microinjection of Ang II into the RVLM can cause the higher elevated amplitude of BP, and the expression of AT_1_R in the spontaneously hypertensive rats (SHR) was higher than in the normotensive rats [13–16]. In contrast, the evidence showed that the central effect of Ang 1–7 had been downregulated in SHR [17, 18].

Previous studies [19–23] have shown that low-intensity exercise training (ExT) is an efficient strategy for patient with hypertension by reducing BP and cardiovascular risk. However, the mechanisms by which ExT improves cardiovascular dysfunction in hypertension have been still unclear. Previous studies often focused on the peripheral mechanism which ExT may act on, such as predominance of endothelium relaxing over contractile factors [24]. Recent evidence suggested that neural plasticity in the central cardiovascular networks can be more important for the beneficial effects of ExT on cardiovascular dysfunctions [25, 26]. It has been known that antihypertension can be caused by blockade of central/peripheral RAS [27]. A previous study further showed that ExT can affect the central RAS via anti-inflammatory cytokines [28]. However, it is still not known whether the ExT-induced benefit on hypertension is dependent or not on balance of the expression/activity of central Ang II and Ang 1–7. Therefore, this study was designed to determine the effect of ExT on the imbalance of the central Ang II and Ang 1–7 in SHR.

## 2. Methods

### 2.1. Animals and General Procedures

Experiments were carried out in eight-week-old male SHR and age-matched normotensive Wistar-Kyoto (WKY) rats purchased from Sino-British SIPPR/BK Laboratory Animal Ltd. (Shanghai, China). All of the procedures in this study were approved by the Second Military Medical University Institutional Animal Care and Use Committee and were performed as per the institutional animal care guidelines.

### 2.2. ExT Protocol and Experimental Design

Male WKY and SHR (eight-week-old) were preselected from their ability to walk on a treadmill (FT-200, Taimen Co., China), and only active rats (≈80%) were used in this study. These selected rats were randomly assigned to the sedentary group (WKY-Sed; SHR-Sed) or the ExT group (WKY-ExT; SHR-ExT). Briefly, the ExT rats ran on a motor-driven treadmill continuously for a period of 12 weeks (5 days per week; 60 min per day at 15–20 m/min), as described in our and other previous studies [29–31]. The training intensity was based on 50–60% of each animal's maximal running speed, which was measured by maximal exercise tests on weeks 0, 6, and 12 (Table 1). Based on a previous study [32], maximal running speed of each group was estimated from graded exercise on treadmill. The test was started at 7.5 m/min with increments of 1.5 m/min every 2 min up to exhaustion. Maximal running speed achieved was considered as the maximal exercise capacity. Training intensity was at 50–60% of each animal's maximal running speed. The sedentary groups were handled at least 3 times every week to become accustomed to the experimental procedures. A noninvasive computerized tail-cuff system (ALC-NIBP, Shanghai Alcott Biotech, Inc., China), as described previously [28], was used to measure BP and HR under conscious condition. The mean arterial BP and heart rate (HR) were measured with volume pressure recording sensor technology, which measures four parameters simultaneously: systolic blood pressure, diastolic blood pressure, mean arterial pressure, and heart pulse rate. BP was measured on three consecutive days, and values were averaged from at least six consecutive cycles. BP was measured at baseline (8 weeks of age) and then every 4 weeks until the end of the training protocol and sedentary period.

After the last exercise session at the age of 20 weeks, animals were allowed at least 24 hr to recover from exercise to minimize alterations in cardiovascular control and further subjected to the following in vivo or in vitro experiments.

### 2.3. Measurement of Citrate Synthase Concentration

Citrate synthase (CS) activity is a validated biomarker for mitochondrial density in skeletal muscle and used as a biochemical marker which undergoes adaptive increase in skeletal muscle with ExT [33]. Soleus muscles from the right leg of the animals were collected, weighted, and stored at −80°C for measurement of citrate synthase. Rat Citrate Synthase ELISA kit (E03876; Shanghai Yunyan Biological Technology Co, China) was used to measure citrate synthase concentration in the whole muscle. Briefly, muscle tissue was homogenized in an extraction buffer and centrifuged at 4°C and an aliquot of supernatants was collected for measuring the enzyme concentration. Reaction was terminated by an addition of a sulfuric acid solution, and the color change was measured spectrophotometrically at a wavelength of 450 nm. The concentration (pg/mg) of citrate synthase in the samples was then determined by comparing the OD of the samples to the standard curve.

### 2.4. General Surgical Procedures and Intracerebroventricular (ICV) Injections

The surgical procedures were carried out as our study described previously [34]. Briefly, rats were anesthetized with urethane (800 mg/kg ip) and *α*-chloralose (40 mg/kg ip), and the trachea was cannulated to facilitate mechanical respiration. The right common carotid artery was catheterized for BP measurement by PowerLab (8SP, AD Instruments). The mean arterial pressure (MAP) and heart rate (HR) were derived from the BP pulse. The vein was cannulated for supplemental anesthesia and drugs. The rat was placed in a stereotaxic frame. A hole in skull surface was drilled for lateral ventricle injections, or the dorsal surface of the medulla was surgically exposed for RVLM microinjections. Body temperature was kept at 37°C by a temperature controller.

The rats were implanted with unilateral guide cannula (21 gauges) for ICV injections of drugs, which was described previously [35, 36]. Coordinates for lateral ventricle injections (−1.0 mm to Bregma; 1.5 mm lateral to midline; and −4.5 mm to the surface of the skull) were based on the rat atlas of Paxinos and Watson [37]. The guide cannula was fixed to the skull with dental acrylic resin. The needle used for injection into the LV was connected by a PE-10 tubing to a 10 *μ*L Hamilton syringe. The needle was carefully inserted into the guide cannula and slow injections were performed. The volume of LV injection each time was 1 *μ*L.

### 2.5. RVLM Microinjection

The procedures for RVLM microinjection were described previously [38]. In brief, microinjections were made from a multibarrel micropipette (20–50 *μ*m diameter) and the coordinates for the RVLM (2.0–2.5 mm rostral and 1.8–2.1 mm lateral to Obex and 2.8–3.2 mm ventral to the dorsal surface of the medulla) were based on the rat atlas [37]. Three-barrel micropipette was connected by a pneumatic picopump (PV820, WPI). The three-barrel micropipette was filled with L-glutamate, tempol, and artificial cerebrospinal fluid (aCSF). The injection volume each time was 100 nL and made over a period of 5–10 s. The aCSF served as vehicle control. The RVLM was chemically identified by a pressor response to L-glutamate (1 nmol) microinjection. The levels of BP and HR were monitored before and after RVLM injection of tempol (1 nmol). At the end of experiments, 2% pontamine sky blue solution was injected into the same site for marking the injection distribution.

### 2.6. Western Blot Analysis

After rats were euthanized with an overdose of pentobarbital sodium (200 mg/kg, ip), the brain tissues including the RVLM, the nucleus of solitary tract (NTS) were punched and collected on coronal sections of brainstem according to the rat atlas [37]. The fixed frozen brainstem was cut into 20 *μ*m thick coronal sections of brainstem by the cryoultramicrotome and punched with a 1 mm internal diameter needle according to the coordinates. As described previously [38], western blot was performed for determining protein levels of ACE, AT_1_R, ACE2, and MasR in the RVLM and NTS. The membrane was probed with anti-ACE (sc-20791, Santa Cruz), anti-AT_1_R (sc-31181, Santa Cruz), anti-ACE2 (sc-20998, Santa Cruz), and anti-MasR (sc-54848, Santa Cruz). The protein bands were visually detected and analyzed. The levels of target proteins were normalized to *α*-tubulin (number T6074, Sigma), which served as a loading control.

### 2.7. High-Performance Liquid Chromatography (HPLC)

As described previously [31], HPLC (model 582 pump, ESA, USA) with electrochemical detection (Model 5300, ESA, USA) was performed to detect 24 h urinary excretion of norepinephrine (NE). Briefly, urinary samples (24 h) were collected and acidified with glacial acetic acid. Dihydroxybenzylamine (Sigma) was used as the internal standard. NE was absorbed onto acid-washed alumina with 3 mol/L tris(hydroxymethyl)aminomethane buffer. After shaking and settlement, NE was extracted with 0.2 M glacial acetic acid (400 *μ*L). Supernatant (40 *μ*L) was injected into HPLC column (reverse phase, ESA 150 × 3.2 mm, 3 *μ*m C18 (P/N 70-0636)), and NE was eluted with mobile phase. The flow rate was set at 0.5 mL/min.

### 2.8. Measurement of Reactive Oxygen Species (ROS) in the RVLM

The level of ROS in the RVLM was evaluated by the oxidative fluorescence dye dihydroethidium (DHE), as described previously [39]. In brief, unfixed frozen brains were cut into 30 *μ*m thick coronal sections of brainstem according to the standard atlas of rat [37]. Brain sections were placed on glass slides. We added the DHE (10 *μ*mol/L) on the brain section directly and did not cover the glass slides with the cover-slips, and then put the glass slides in a light-protected humidified chamber at 37°C for 30 min. The oxidative fluorescence intensity was detected at 585 nm wave length by a laser scanning confocal imaging system (Leica SP5). The average fluorescent intensities were evaluated by Leica software and used for image quantification. This software was designed with the auto model to calculate the fluorescence intensity in the chosen areas.

### 2.9. Statistical Analysis

All data were presented as mean ± SEM. Two-way ANOVA with repeated measures was used to assess the efficacy of ExT during the ExT. Student's *t*-test or two-way ANOVA followed by Newman-Keuls post hoc test was used to assess the differences between groups (WKY and SHR) and conditions (Sed and ExT). Differences with a *P* < 0.05 were considered significant.

## 3. Results

### 3.1. Assessment of ExT Efficacy

**Figure 1:**
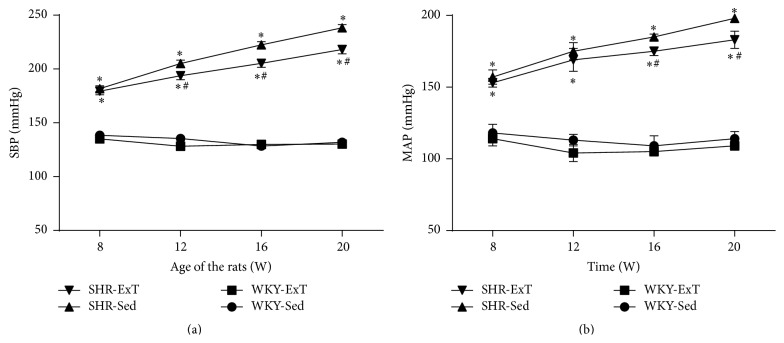
Time course of systolic blood pressure (a) and mean arterial pressure (b) in sedentary or exercised WKY and SHR groups. The values for blood pressure in conscious rats were measured by tail-cuff method. Blood pressure has already lowered in SHR-ExT compared with SHR-Sed rats from 8th week of ExT (at 16 weeks of age). *n* = 15 in each group. ^*∗*^ *P* < 0.05 versus WKY-Sed; ^#^ *P* < 0.05 versus SHR-Sed. *P* value for groups was <0.001, *P* value for time was <0.001, and group *x* time interaction was <0.001.

At the beginning of ExT (rats at age of 8 weeks), MAP of SHR-Sed (157 ± 5.1 mmHg) in conscious condition was significantly higher (*P* < 0.05, *n* = 15) than WKY-Sed (118 ± 6.2 mmHg) and remained the higher levels for the time of the study. After 12 wk period of ExT, MAP was significantly lower (*P* < 0.05, *n* = 15) in SHR-ExT (183 ± 6.2 mmHg) than in SHR-Sed (198 ± 3.2 mmHg). However, WKY rats showed no change of BP following ExT treatment (Figure 1). As shown in Table 2, several values were measured for effects of ExT at the end of Sed or ExT protocol (at age of 20 weeks). Body weight in ExT groups was significantly reduced compared with Sed groups. Soleus muscle weight was significantly increased in ExT groups compared to Sed groups. Twenty-four-hour urinary excretion of NE level was higher in SHR-Sed than in WKY-Sed, whereas it was significantly reduced following ExT protocol. In additional, we confirmed that the concentration of citrate synthase, a marker of ExT efficacy, in soleus muscle was significantly higher in ExT than in Sed rats.

### 3.2. ExT Modulates RAS Components in the RVLM of SHR

**Figure 2:**
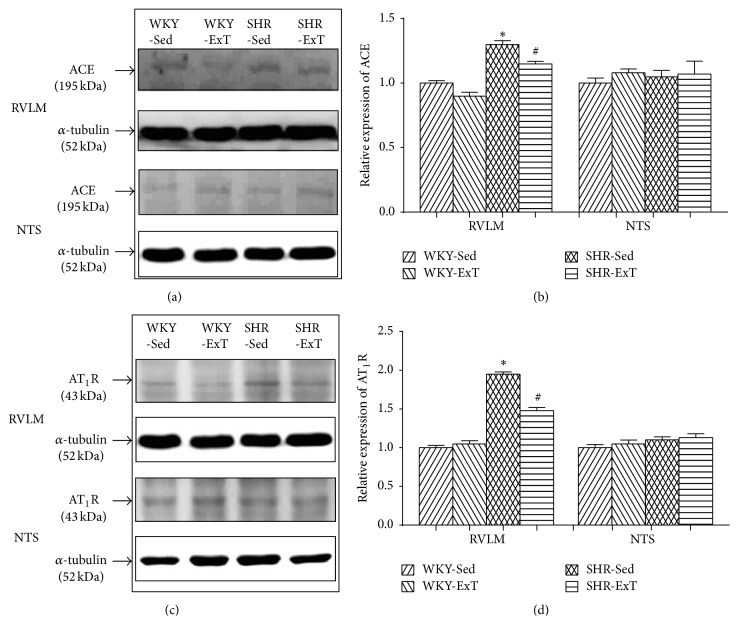
Representative western blot (a, c) and densitometric analysis (b, d) of ACE and AT_1_R in the RVLM and NTS of sedentary or exercised WKY and SHR groups. *n* = 5 in each group. ^*∗*^ *P* < 0.05 versus WKY-Sed; ^#^ *P* < 0.05 versus SHR-Sed. As to AT_1_R in the RVLM, *P* value for WKY-SHR was 0.0006, *P* value for Sed-ExT was 0.0616, and *P* value for cross interactions was 0.0177; as to ACE in the RVLM, *P* value for WKY-SHR was <0.0001, *P* value for Sed-ExT was 0.0045, and *P* value for cross interactions was 0.1937; as to AT_1_R in the NTS, *P* value for WKY-SHR was 0.3501, *P* value for Sed-ExT was 0.7997, and *P* value for cross interactions was 0.8495; as to ACE in the NTS, *P* value for WKY-SHR was 0.5408, *P* value for Sed-ExT was 0.4906, and *P* value for cross interactions was 0.9744.

**Figure 3:**
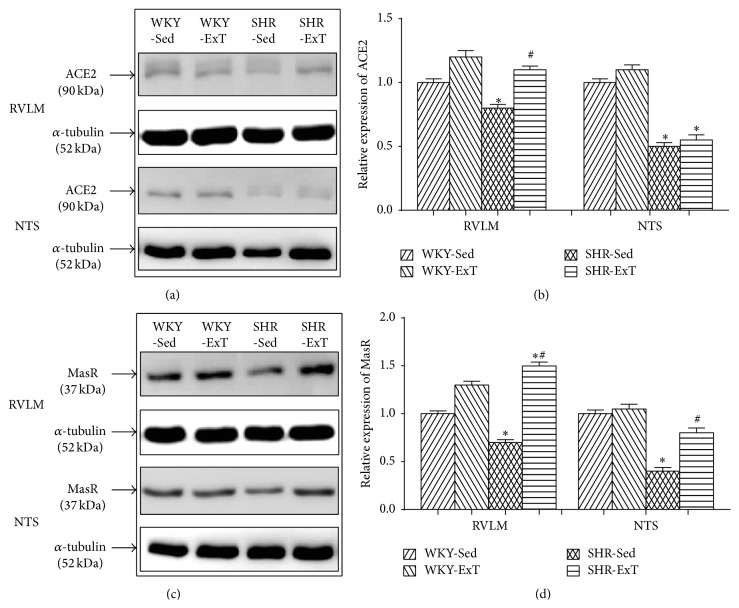
Representative western blot (a, c) and densitometric analysis (b, d) of ACE2 and MasR in the RVLM and NTS of sedentary or exercised WKY and SHR groups. *n* = 5 in each group. ^*∗*^ *P* < 0.05 versus WKY-Sed; ^#^ *P* < 0.05 versus SHR-Sed. As to ACE2 in the RVLM, *P* value for WKY-SHR was <0.0001, *P* value for Sed-ExT was <0.0001, and *P* value for cross interactions was 0.0002; as to MasR in the RVLM, *P* value for WKY-SHR was 0.1891, *P* value for Sed-ExT was <0.0001, and *P* value for cross interactions was 0.0003; as to ACE2 in the NTS, *P* value for WKY-SHR was 0.0002, *P* value for Sed-ExT was 0.9242, and *P* value for cross interactions was 0.8496; as to MasR in the NTS, *P* value for WKY-SHR was 0.0075, *P* value for Sed-ExT was 0.0231, and *P* value for cross interactions was 0.0612.

To determine whether ExT modulates the components of RAS, we examined the protein levels of ACE, AT_1_R, ACE2, and MasR in the RVLM and the NTS. As shown in Figure 2, the protein expressions of ACE and AT_1_R in the RVLM were significantly higher in SHR-Sed compared with WKY-Sed, which were downregulated following ExT treatment. However, these two proteins showed no differences in the NTS of rats with or without ExT. In additional, the protein expressions of ACE2 and MasR levels in the RVLM and NTS were significantly reduced in SHR-Sed compared with WKY-Sed (Figure 3). ExT significantly increased ACE2 and MasR expression in the RVLM of SHR. In the NTS, interestingly, only MasR expression was significantly increased in SHR with ExT.

### 3.3. ExT Improves Central Functionality of ACE-Ang II-AT_1_R Axis and ACE2-Ang-1–7-MasR Axis in SHR

**Figure 4:**
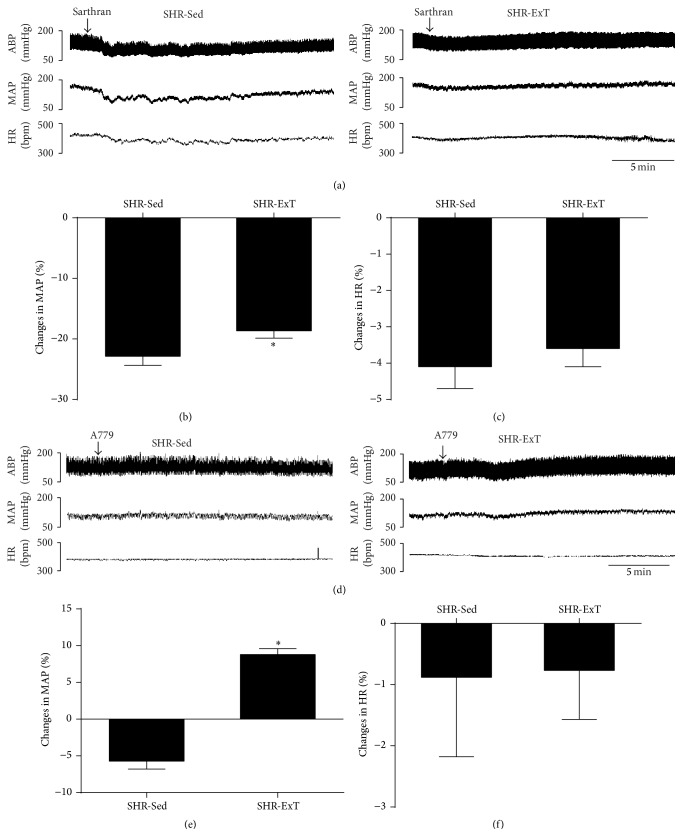
Representative original recordings of effects of ICV infusion of sarthran (15 nmol) or A779 (500 pmol) on cardiovascular activities in sedentary or exercised SHR groups (a, d). ABP: arterial blood pressure; MAP: mean arterial pressure; bpm: beats/min. Percent changes in mean arterial pressure (b, e) and HR (c, f) induced by ICV infusion of sarthran (15 nmol) or A779 (500 pmol) in the two groups. *n* = 5 in each group. ^*∗*^ *P* < 0.05 versus SHR-Sed.

To investigate the influence of ExT on central functionality of RAS in SHR, we examined the cardiovascular response to acute ICV injection of the Ang II antagonist sarthran and the Ang 1–7 antagonist A779. As shown in Figure 4, the depressor response evoked by ICV injection of sarthran (15 nmol) was significantly attenuated (−40 ± 3.4 versus −28 ± 3.8 mmHg) in SHR with ExT treatment. It was interesting that the depressor effect (−10 ± 3.5 mmHg) of ICV injection of A779 (500 pmol) was reversed to a pressor response (13.2 ± 2.5 mmHg) in SHR following ExT treatment. However, there was no difference in HR between SHR-Sed and SHR-ExT.

### 3.4. ExT Attenuated ROS Production in the RVLM of SHR

**Figure 5:**
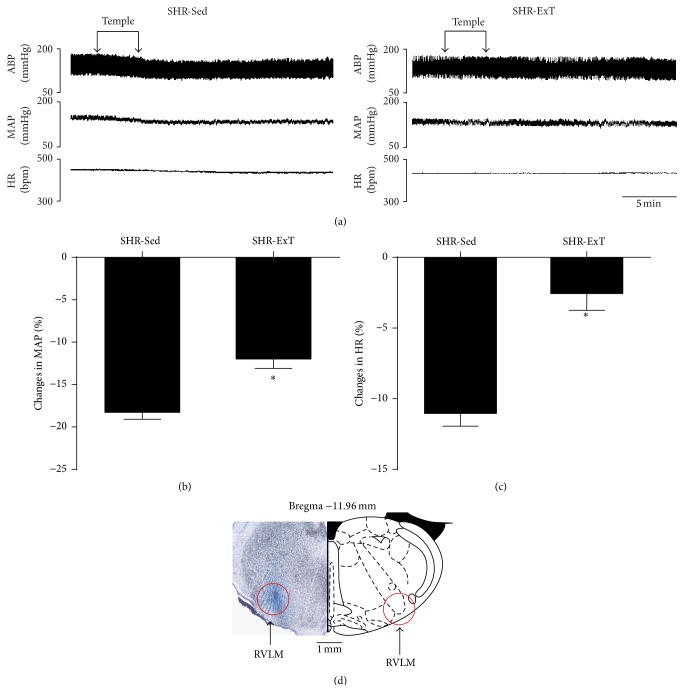
(a) Representative original recordings of effects of the SOD mimic tempol (1 nmol) bilaterally injected into the RVLM on BP and HR in sedentary or exercised spontaneously hypertensive rat groups. ABP: arterial blood pressure; MAP: mean arterial pressure; bpm: beats/min. Percent changes in mean arterial pressure (b) and HR (c) induced by bilateral microinjection of tempol into the RVLM. (d) Distribution of the maker sky blue within the brain section. *n* = 5 in each group. ^*∗*^ *P* < 0.05 versus SHR-Sed.

**Figure 6:**
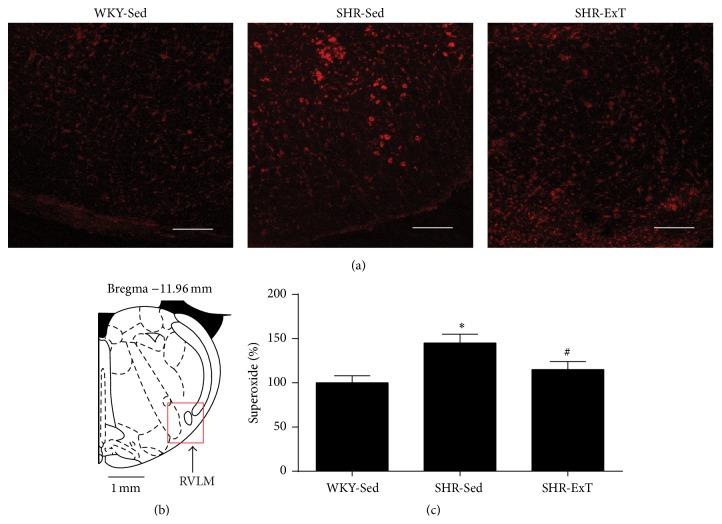
Effects of exercise training on level of ROS in the RVLM from SHR. (a) Representative DHE fluorescence staining images (red color) ROS content in the RVLM of sedentary or exercised SHR and sedentary WKY groups. (b) A red square in a coronary drawing of rat standard atlas of RVLM. (c) Bar graphs show ROS content obtained from DHE fluorescence intensities in the RVLM of SHR. The value of DHE fluorescence intensities in the sedentary WKY was normalized as 100%. *n* = 5 per group. ^*∗*^ *P* < 0.05 versus WKY-Sed, ^#^ *P* < 0.05 versus SHR-Sed. Scale bars = 200 *μ*m.

As shown in Figure 5, microinjection of the SOD mimic tempol (1 nmol) into the RVLM produced a greater depressor response in SHR-Sed, which was significantly attenuated in SHR with ExT treatment. DHE staining showed that the production of ROS in the RVLM was significantly higher in SHR-Sed than in WKY-Sed, which was significantly decreased in SHR-ExT (Figure 6).

## 4. Discussion

The major observations of this study are that (1) ExT significantly reduces BP and sympathetic activity in SHR; (2) ExT treatment improves the functionality of central ACE-Ang II-AT_1_R and ACE2-Ang-1–7-MasR in SHR; and (3) ExT significantly reduced the level of ROS in the RVLM of SHR. On basis of these results, we conclude that ExT improves central RAS and decreases oxidative stress in the RVLM of SHR.

ExT, a part of lifestyle modification, has been recognized as an important strategy for antihypertension [40, 41]. However, the exact mechanism by which ExT improves the cardiovascular dysfunctions in hypertension is still unclear. It has been documented that the low-intensity ExT effectively decreases BP in hypertension [42]. In this work, we tested the maximum ExT capacity (velocity) at the beginning of the protocol and further determined the ExT intensity at weeks 6 and 12, which was controlled at 50–60% of maximal ExT capacity according to previous studies [31]. Our study showed that the maximal running speed of SHR was faster than the WKY rats, which was completely consistent with a previous study [43]. The reason for high exercise capacity in the SHR is not clear. It is possible that the enhanced cardiac output, blood pressure, and sympathetic activity in the SHR are attributed to the higher exercise capacity than WKY rats. We found that soleus muscle weight and citrate synthase concentration were significantly increased in ExT groups compared to Sed groups, whereas body weight was reduced in ExT group compared to Sed groups. We also confirmed that ExT significantly lowers BP in SHR but in WKY, as described previously [44]. It is notable that the data of BP was somewhat higher in conscious animals (measured by tail-cuff method) than in anesthetized rats (measured by arterial cannula). This high level of BP in conscious rats may have resulted from the stress induced by the tail-cuff method. Additionally, urinary excretion of NE was significantly decreased in SHR after treatment of ExT. It may be a limitation that the direct recording of sympathetic nerve activity in rats was not performed, which would be more specific measurement. However, elevation of NE can be partly correlated with the enhanced cardiovascular sympathetic activity. Collectively, the current data confirmed the efficacy of ExT to BP and sympathetic activity in SHR.

Previous studies showed that ExT improved the cardiovascular function in hypertension via peripheral and central pathways [24, 26]. For example, ExT can enhance the release of nitric oxide which had been downregulated in hypertension [24]. However, it is also suggested that the central effect of the ExT plays an important role in antihypertensive mechanism [25, 26, 43, 45]. For example, ExT effectively improves abnormalities in the cardiovascular reflex (e.g., baroreflex, chemoreflex). In hypertension, the function of central Ang II is upregulated, whereas central Ang 1–7 is downregulated [16]. Previous study also showed that ExT can change components of RAS via anti-inflammatory cytokines [28]. This study focused on balance between pro- and anti-inflammatory cytokines in the brain of SHR affected by ExT, but the effect of ExT on the functional change of endogenous RAS was still unknown. In order to detect the effect of ExT on RAS involved in cardiovascular regulation in the whole central nervous system, the agents of A779 or sarthran were injected into the lateral ventricles in this study. Because the RVLM is a key region involved in control of resting blood pressure and sympathetic outflow, it was further subjected for detecting the protein levels of ACE, AT_1_R, ACE2, and MasR in response to ExT, which is helpful for determining the possible mechanism responsible for the effect of ExT on central cardiovascular regulation. However, BP and HR in WKY groups have not been changed in response to ExT treatment. Moreover, ExT did not change the expression levels of RAS components in the RVLM of WKY groups. Our data suggested that ExT is capable of improving the balance between Ang II and Ang 1–7 in the brain of SHR. The data from in vivo experiments showed that ExT decreased the cardiovascular response to central blockade of Ang II in SHR. The change in BP evoked by acute injection of Ang 1–7 seems to be similar to Ang II, while the response sensitivity to central Ang 1–7 is increased in SHR following ExT. This data reveals that ExT increases the MasR-mediated actions in central nervous system. ExT reduces the depressor response to central blockade of AT_1_R with sarthran in SHR, suggesting that ExT decreases the endogenous Ang II via downregulating ACE. Additionally, blockade of the MasR with A779 showed that the cardiovascular response to the endogenous Ang 1–7 was more sensitive in SHR with ExT, suggesting that the activity of the MasR is upregulated by ExT treatment. Previous studies show that acute injection of exogenous Ang II and Ang 1–7 produced a similar effect on BP in the RVLM [46] and NTS [47]. Interestingly, increase in endogenous Ang 1–7 by overexpression of ACE2 in the RVLM produced a long-term decrease in BP in hypertensive rats [48]. Acute or chronic increase in Ang 1–7 may present different effect on cardiovascular regulation. So, the significance of Ang 1–7 in the brain area in cardiovascular regulation needs to be further determined. In this study, the ExT-induced enhancement in Ang-1–7/MasR axis may have resulted from chronic upregulation of ACE2. Because comparison of central cardiovascular response to RAS components between SHR and WKY has widely been reported, only SHR rats with or without ExT were performed to in vivo functional experiments in the present study. This may be a limitation in this study. However, we had verified the change of RAS in WKY and SHR groups via the western blot showing the change of protein expression of RAS components.

Although ExT adjusts the central RAS-mediated cardiovascular effects, it is not clear which cardiovascular region is involved in the effect of ExT on central RAS in hypertension. In view of the importance of the NTS and RVLM in regulating cardiovascular function [49, 50], they are subjected to further experiments. It is reported that ExT downregulates the expression of angiotensinogen mRNA but has no effect on AT_1_R in the NTS [43]. We also confirmed that ExT had no effect on AT_1_R expression in the NTS of SHR. In the RVLM, ExT significantly reduced ACE and AT_1_R expression and increased ACE2 and MasR expression in the RVLM of SHR. It is worth to concern the specificity of commercially available antibodies for the AT_1_R [51]. In this work, however, we confirmed that functional state of central AT_1_R by ExT was reduced by observation of BP and HR changes in response to central injection of sarthran. These data indicate that the RVLM may be a main region involved in the effect of ExT on central RAS in hypertension.

In the RVLM, oxidative stress originated from RAS contributes to high levels of BP in hypertension. We first confirm that the decrease in BP evoked by injection of the SOD mimic tempol into the RVLM is attenuated by ExT in SHR, suggesting that ExT effectively reduced the ROS-mediated cardiovascular function in SHR. This is consistent with a previous study [52]. Importantly, we directly detected the level of ROS by DHE probe in the RVLM. We further confirmed that the level of ROS within the RVLM was higher in SHR than in WKY, which was attenuated by ExT. It may be a limitation that the ROS level between WKY-Sed and WKY-ExT was not further compared. The mechanism responsible for the ExT-mediated antioxidative stress in the RVLM of hypertension is not clear. It is reported that activation of the AT_1_R-mediated NADPH oxidase plays an important role in ROS production in hypertension [53]. Therefore, it is possible that downregulation of ACE-Ang II- AT_1_R axis by ExT is an important pathway for antioxidative stress in the RVLM. Additionally, upregulation of ACE2-Ang-1–7-MasR axis by ExT is also involved in reducing oxidative stress in the RVLM.

In summary, the chronic ExT improves the cardiovascular function in hypertension. However, the antihypertensive mechanism of ExT is fully not understood. Here we provide new insight into the signaling mechanism by which ExT is capable of improving the balance between Ang II and Ang 1–7 in the RVLM of hypertensive rats.

## Figures and Tables

**Table 1:** Maximal running speeds in ExT groups.

	Maximal speeds (km/h)			
	*n*	Week 0	Week 6	Week 12
WKY-ExT	15	1.33 ± 0.07	1.76 ± 0.05^*∗*^	2.21 ± 0.06^*∗*^
SHR-ExT	15	1.87 ± 0.06^†^	2.04 ± 0.08^*∗*†^	2.43 ± 0.07^*∗*†^

Data are mean ± SE. ^*∗*^ *P* < 0.05 versus week 0. ^†^ *P* < 0.05 versus WKY-ExT.

**Table 2:** Measurements of parameters for determining efficacy of ExT.

Parameters	*n*	WKY-Sed	WKY-ExT	SHR-Sed	SHR-ExT
Body weight (BW, g)	15	320 ± 5	292 ± 4^*∗*^	305 ± 5	280 ± 4^†^
SMW (mg)	15	110 ± 3	136 ± 4^*∗*^	105 ± 5	137 ± 4^†^
SMW/BW (mg/g)	15	0.34 ± 0.01	0.47 ± 0.01^*∗*^	0.34 ± 0.01	0.49 ± 0.01^†^
CS in SMW (pg/mg)	15	672 ± 25	790 ± 35^*∗*^	685 ± 25	778 ± 30^†^
MAP (mmHg)	10	122 ± 3	116 ± 6	175 ± 5^*∗*^	150 ± 5^*∗*†^
HR (beats/min)	10	397 ± 8	352 ± 9^*∗*^	453 ± 8^*∗*^	392 ± 7^†^
NE in 24 h urine (*μ*g)	10	0.41 ± 0.03	0.39 ± 0.05	0.71 ± 0.05^*∗*^	0.55 ± 0.04^*∗*†^

*n*: number of animals; MAP: mean arterial pressure; SMW: soleus muscle wet weight; CS: citrate synthase; MAP: mean arterial pressure; NE: norepinephrine; WKY-Sed: Wistar-Kyoto-sedentary; SHR-Sed: spontaneously hypertensive rat-sedentary; WKY-ExT: Wistar-Kyoto-exercise training; SHR-ExT: spontaneously hypertensive rat-exercise training. Data are mean ± SE. Values for MAP and HR were obtained in anaesthetized rats. MAP was measured by catheterizing femoral artery. ^*∗*^ *P* < 0.05 versus WKY-Sed. ^†^ *P* < 0.05 versus SHR-Sed.
